# Human Knowledge,—Natural and Supernatural

**Published:** 1861-04

**Authors:** B. P. Aydelott

**Affiliations:** President of the Board of Trustees


THE
DENTAL REGISTER OF THE WEST.

Vol. XV.] APRIL, 1861. [No. 4.

### Original Essays and Communications.

### HUMAN KNOWLEDGE, NATURAL AND SUPERNATURAL—ITS CHANNELS AND FOUNDATION.

AN ADDRESS,

*Delivered at the Commencement of the Ohio College of Dental Surgery,* 1861.

BY B. P. AYDELOTT, D. D.,
President of the Board of Trustees.

Our subject involves the profoundest depths of metaphysics, and yet we shall not be metaphysical, in the ordinary sense of the term; at least, we will endeavor not to be so.

We do not feel obligated, therefore, on the present occasion, by the precise definitions of metaphysical or psychological science; neither are we bound to use only its terminology or language; or indeed to have recourse to these at all where we can get along as well without them. We will try to walk only in the light of common sense, and employ just such words as plain, intelligent people use in every-day intercourse.

Our subject is—Human Knowlege, natural and supernatural—its channels and foundation.

By *human knowledge* we mean every thing which man,—not angels,—but man can know about the Universe around him, and about himself. And by the *foundation* of this knowledge we intend that *in man* on which all his knowledge must ultimately rest.

Let it be premised also that, in what follows, we shall speak of man chiefly as an intelligent creature: and yet our subject would be incomplete, neither could the discussion of it be altogether satisfactory, if we did not also, before we closed, bring to view man's *great moral* relation. To proceed to our purpose:

We ask your attention, then, first to—

I. The Channels of Knowledge. What are these? They are first—

1. The *senses.* These are commonly called the external senses, the animal senses, or the five senses. Some psychologists have indeed contended that all the senses may by analysis be reduced to one, that of feeling; and others, on the contrary, have proposed to add several more to the generally recognized five-fold division of the senses. We shall not here enter into these nice distinctions; but confine ourselves to the popular understanding of the term senses.

By these senses, then, viz: that of sight, hearing, touch, taste, and smell, we take cognizance of all we can know of the phenomena of matter, or of the external world—that is, all *outside of the mind.*

Every thing that is pleasing, beautiful, and sublime in prospect, every thing that is agreeable and ravishing in sound; every thing that is soothing or pleasurable in feeling; and every thing calculated to gratify in taste and odor;—or to produce the opposite feelings—all these we receive through the senses.

Furthermore,—all those facts on which rest the Natural Sciences, various and vastly comprehensive as these sciences are; those facts also on which chemistry and the healing art are founded, and those which furnish the basis of astronomy; and the almost numberless facts which go to the building up of natural philosophy generally:—all these facts are made known to us by the senses.

And if there is anything else in man's knowledge or science derived from a material basis, this also must be taken into account.

The almost illimitable field thus spread before the senses, and therefore the vast importance of the senses as channels of knowledge, must, even from this brief and hurried glance, be seen and felt.

It is manifest also that without these senses each one of us would be an isolated being,—ignorant, totally ignorant of all around him, and of all within him too,—a vitalized organism —a merely animated statue of bones and flesh, soon to fall into ruins. It is through the senses man first awakens up to himself and the world about him.

A second channel of knowledge is—

2. The power of *intuition.*

At the very first glance of certain facts and truths, as presented in the mind, we may instantly arrive at the knowledge of other facts and truths. The faculty by which this attainment is made has been called by a variety of names, as intuition, suggestion, simple suggestion, judgment, common sense, consciousness, etc. The knowledge we thus get does not come to us through the senses, neither is it the deductions of reason. It is discerned by a mere glance of the mind, and is accompanied by as clear a conviction of its truth, as is the information of our senses, or the conclusions of our reason. Men ask no proof of it; it is self-evident.

The knowledge we thus get has been called by a great variety of names, as intuitions, intuitive judgments, intuitive convictions, native convictions, spontaneous convictions, necessary cognitions, necessary truths, primary ideas, fundamental laws of human belief, dictates of common sense, etc. A moment's reflection will show us the importance of this knowledge.

It is evident that all progress in knowledge by a created, intelligent being, like man, implies a beginning, a starting point. This he must assume at the commencement of his career. And it is equally clear also that, if two such beings wish to argue any subject, they must begin somewhere, and that place of beginning must be assumed by both parties, however far back they may have to go before they find it. From this point, then, assumed and granted, both parties must start, or forever stand still.

In this way we all,—whether as individuals commencing our progress, or as parties about to discuss any matter,—we must have a fixed point of departure. Now, these necessary cognitions, or dictates of common sense, or fundamental laws of human belief, are just the things taken for granted in all such progress.

For example:—in mathematics, *things which are equal to the same thing are equal to each other. If equals be added to equals, the wholes will be equal. The whole is greater than any of its parts. The whole is equal to the sum of all its parts.* We must see and acknowledge the truth of these and other axioms at the very start, or we can not advance a single step in mathematics.

So also in ethics or moral philosophy: That *there is a natural and eternal distinction between right and wrong.* That *we are moral and responsible beings.* These primary truths, to name no more, must be assumed, or the science of morals is impossible to us.

Again: the intuitive judgment brought to bear also upon the state and the acts of the mind itself, furnishes us ultimately with all the fundamental facts or truths of psychology or intellectual philosophy.

And in the physical sciences, too, all our progress in every branch of knowledge which relates to the material world, as natural philosophy, the natural sciences, chemistry, medicine. etc.—in all these we must see and allow at the outset certain great fundamental truths, as, for example, that *every effect must have an adequate cause.* And again: *The uniformity of nature;* that is, *the laws which have hitherto governed the material world will continue so to do hereafter.* If any one deny these and similar truths, he can make no advance whatever in natural philosophy, or in any other department of physical science. Without these intuitions he has neither light to guide him, nor capacity to walk.

In a word, intuitive judgments, or the fundamental laws of human belief, lie at the foundation of all science, and are involved in every step of our progress.

Such, then, is our indebtedness to intuition as a channel of knowledge.

A very interesting and instructive incident, illustrating this subject is related of the celebrated Dr. Beattie, author of "THE MINSTREL," etc. He wished to learn by experiment whether the truth that every effect must have an adequate cause, was a spontaneous conviction of the mind. To ascertain this, he sowed some lettuce seed in his garden in such a way as to make the three letters J. H. B., the initials of his son's name, then a very small child, James Hey Beattie, afterwards a co-professor with his father in the University.

When the seed had sprung up, the Doctor took little James with him to walk in the garden. The child ran hither and thither, attracted by different objects, but soon came quickly back to his father with an expression of wonder in his face, and urged him to come and see something he had found out. The Doctor suffered himself to be led to the spot where the lettuce was growing, and purposely looked upon it with an air of indifference. The child, disappointed and astonished at this, asked his father to tell him how it was so. The Doctor replied, "Did it not come of itself?" Little James dropped his head for a moment in much seeming perplexity, but quickly answered—"*No, father! It couldn't come of itself; some body must have put it there.*"

The experiment was successful. Even a child could see that every effect must have an adequate cause.

But we proceed to another means of knowledge. It is,

3. The *reason*, or discursive faculty.

We use the term *Reason* here in its most comprehensive import, to signify that power of mind by which we discover new truths from a comparison of ideas, of whatever length the process may be,—a single step or ten thousand such.

Writers on Psychology, or the Philosophy of the Human

Mind, have analyzed this capacity into two or more elementary powers; but it is not needful for us here to make such distinctions. It is sufficient for us to notice the fact that, through this capacity of reason, or the discursive faculty, we arrive at a vast amount of knowledge; and that there is no possibility of setting bounds to our progress in this field. To our reason do we owe all our sciences—intellectual, physical, moral, and mathematical. These come to us simply through this channel. Without the discursive faculty, science would be impossible to us. It is, therefore, one of the principal distinctions between man and the lower animals. He is capable of science; they can never attain to it.

The fourth and last channel of knowledge is—

4. The *teaching* of others.

There are two things here very remarkable; the first is, that nearly all our knowledge we get in this way; and the second thing to be noted is, that very few seem to be sensible of this their indebtedness.

Let any one but try the experiment with himself; let him take as full and complete a survey as he can of his knowledge, and he will find that by far the larger part of this he has derived from others. They communicated it, he only received it. He has sat at the feet of the living teacher, or studied the volumes of authors, many of whom had gone to their graves long before he was in being.

We will not here point in proof of this to the history of the past; for this, valuable and voluminous as it is, we manifestly get altogether from others. We know nothing of the events of history, and we can know nothing of them of ourselves. These facts have all transpired, and scarcely any thing remains but the record of then for our instruction.

Let any one, however, look at his geography, and see to what a very small compass it would be reduced were he obliged to cast out all he had not observed for himself. Verily, the geography of the most of us would be limited to a very partial knowledge of a few cities, towns, and villages of our own land, and some of the roads between these, and the narrow tract of country surrounding them.

And how contracted must his—now perhaps wide—sweep of astronomy become by such a process! How few of us—even the most intelligent of us—have observed for ourselves the moons of Jupiter, or seen the great planet Neptune, or counted the tens of thousands of fixed stars and nebulæ scattered through the vast fields of space, which the telescope has brought to view.

And to name no more—to what exceedingly narrow limits would our chemistry, our natural philosophy, and our knowledge of the physical sciences generally be reduced, were every thing to be blotted out of our minds which we had not observed for ourselves. How few of us have ever seen the one tenth, or even the one hundreth part of the facts and experiments on which these sciences rest. But all these important facts and experiments and the vast and precious systems of science built upon them—all these we must give up, if we shut out the teaching of others, and confine ourselves to the limits of our own observation.

Having thus taken a quite general and very rapid survey of man's knowledge, and pointed out the channels through which it all comes to him; we are now prepared, secondly, for the deeply interesting and very important question—

II. On what does all this vast and continually enlarging fabric of human knowledge REST?

We answer *faith*, simply FAITH. Had we no capacity of trust, no ability of confidence, no power of reliance,—in a word, had we no *faith* in our senses, our intuitive judgment, our discursive faculty, and the teaching of others; or could our faith in the testimony of these fail, where would all our knowledge be? That vast edifice which the human family has been perseveringly engaged in building up ever since the world began—our natural sciences, our ethics, our mathematics, our astronomy, our natural philosophy—in a word, all our knowledge, intellectual, moral, and physical,—where would this all be, without faith? It must instantly vanish like the baseless fabric of a dream. Aye, and the very dream itself would speedily disappear. And in thus annihilating all outside of ourselves—the universe about us—we should annihilate ourselves also. And hence, alike, from this, the deepest abyss of Scepticism, and from the cloud capped heights of Transcendentalism, would the insane shout go up, GREAT IS NOTHING! Without faith in our faculties, such must be the dreary end of all our endeavors, all our progress, all our aspirations. Without faith there could be no knowledge, no reality to us.

But, thank God, we can not come to this! Here and there indeed, a dreamy metaphysician in his closet, or a trifler with God's truth, or a wilfully wicked, corrupt man may argue himself into intellectual scepticism; but the great mass of mankind have not so destroyed their mental sanity. They have enough yet left of health of mind to trust their senses and to hold fast the dictates of their intuitive judgments, and confidently to rely upon the deductions of their understandings, and to place a reasonable and generous reliance upon the teachings of others. And thus has the world hitherto gone on, *by faith*, growing in knowledge, and is now advancing with an ever-accelerating speed in all departments of a true and safe science,—continually aspiring towards a glorious consummation. And this onward march can not be arrested. Why? Just because the great mass of men will have *faith* in themselves; they can not be persuaded out of their senses. And they will ever regard, as they have hitherto regarded, the few who profess such morbid doubts as dishonest men to be abhorred; or they will pity them as the victims of a foolish and ruinous hallucination.

Such, then, is the ultimate foundation in himself of all man's knowledge, natural and supernatural.

But it may here be asked,—have none ever gone further than this? Is there nothing *without*, on which man may place his foot with as healthful and unfailing a trust as on that *inner* foundation of which we have just spoken?

But before answering this question, we beg leave to remind you of our statement at the outset, that we would purposely avoid psychological precision of definition and terminology as far as possible. Hence, when we inquired above, whether there was another foundation on which we might rest beside *faith in our faculties*, we do not wish to be strictly understood. For, in truth, there can be no other basis of human knowledge than this faith—all we know must ultimately rest upon it.

But let it be considered that as we advance in every department of knowledge and true science, each attainment thus made throws back such fresh light upon the foundation as makes its reasonableness and security more and more manifest. Hence, our very attainments seem almost another basis of knowledge, and for all practical purposes, they do serve this end. And, therefore, intelligent, sound-minded men, and especially profoundly learned men, are rarely betrayed into scepticism.

But such has hitherto been the state of the world, that a high degree of intelligence has not been universal; and great scholars have always been very rare in comparison with the mass. Hence, men generally have not been able to bring much intellectual culture,—much less great scholarship,—to the aid of their faculties and of faith in these.

But not so in respect to the argument of which we are now about to speak. It is an argument plain and obvious even to the simplest and most unlettered minds; and though perhaps such have rarely thrown it into logical form, they have for the most part always seen and felt its force. It is an argument—be it here also observed—which rests exclusively on man's *great moral relation;* and the whole tendency of which is to bring that relation most vividly, practically and permanently into contact with all the susceptibilities and powers of man's nature.

We return, then, and repeat the question, Is there nothing *without* on which man may place his foot with as healthful and unfailing a trust as on that *inner* foundation of which we have spoken? We answer, there is; and in as few words as possible will we endeavor to bring plainly to view this other and higher *Rock* on which it is alike our duty and our privilege to rest.

We are not called upon here to say *how* it is that the world has become possessed of the deep-seated, indelible conviction that there is a God—a Power above it. Some have ascribed this to the exercise of man's own faculties upon the evidences spread all around him of God's wisdom, power and goodness in his works; others have traced it to an original divine revelation, handed down through successive generations by tradition. However this be, the fact is certain, that no nation or tribe of men, however remote, or deeply sunk in ignorance and barbarism, has yet been found without some ideas of a Divine Being—a Power above them; and their religious observances towards this power constitute their superstition, which they must have where they are without true religion. Indeed, superstition, of one shape or another, is the religion of all ignorant men.* But passing this.

We who live in Christian countries, and with the Bible in our hands, have clearly revealed to us God's being and character,—the eternal, almighty, infinitely wise, just, good, and holy Creator and Preserver of all things, the righteous Judge and sovereign Ruler of heaven and of earth. And these teachings of revelation we see abundantly confirmed by every day's observation of things within and without us, and by every advance we make in the sciences.

---

"Pass over the earth," said Plutarch, "you may discover cities without walls, without literature, without monarchs, without palaces or wealth, where the theater and the school are not known; but no man ever saw a city without temples and gods, where prayers, and oaths, and oracles and sacrifices were not used for obtaining pardon or averting evil."

Here and there, indeed, a modern traveler or voyager has reported some obscure, savage people who have no idea of a God; but, subsequently, larger and more careful examination has always corrected this report,—shown it to be hasty and unfounded. So that the remark of the philosophic biographer is found to be as true now, as it was when he penned it nearly two thousand years ago—"No man ever saw a city without temples and gods," etc.

Now, with this conviction of a God pervading our every faculty, and going down into the deepest recesses of our bosoms, and accompanying us in all our progress, we can, and we daily do, frame an argument for the certainty of human knowledge—an argument short indeed, and yet so clear, so overwhelming in its demonstration, that, once we have seen it, we can not get rid of it, till we deny every thing that is sensible, and good and noble in our own nature. The argument is this:

God gave me not only my being, but the very constitution of my being—all my faculties, intellectual and moral; and I must believe these,—it is in the highest degree reasonable and right to believe them. If I do not, what follows? what follows? I make *my nature a lie, and God the author of it!*

But this is a conclusion from which every religious, nay, every reasonable mind instantly revolts. All the light that is in us, and all our moral instincts cry out, God is a God of truth, and can not lie! His throne is built upon truth. All his laws uphold truth. His government protects and encourages truth, and pours contempt upon falsehood, and holds out never-ending woe to all liars. The whole course of his providence also tends to establish the lip of truth and bring to naught the counsels of liars. Look where I will, truth shines out in all God's works and ways. To admit, therefore, for a single moment that my nature is a lie, and God the author of it, is either desperate madness, or a monstrous sin not only against God, but against myself and the whole constitution of things!

We have, then, God's guaranty for the certainty of human knowledge. So sure as the Lord reigneth, truth is eternal, and we do well to rely upon the faculties He has given us. These faculties are his *witnesses;* and, if properly questioned, can never deceive us. Hence, their testimony has been uniformly received by the sensible and the good everywhere and at all times; and in *this faith* have they not found great reward?

Here and there, indeed, have been seen a few dreamy closet speculators, and a few proud, disappointed spirits, and a few selfish, designing men, and a few men of corrupt ways, and a few desperately bad men, who have professed to doubt every thing, and poured contempt over all human knowledge. But does not the daily conduct of such men always show them to be "vain disputers,"—insincere, hypocritical, sinners not only against the common sense of their fellow-men and the truth of God, but against their own convictions also? For have they not ever lived as other men do?—kept out of danger?—taken care of their own interests?—sought out and enjoyed the good things of life? and made loud complaints, too, when their rights were invaded? In a word, have they not lived, and spoken and acted just as all those do who hold fast to the certainty of knowledge and the reality of things? Thus do the daily lives of such men proclaim the falsehood of their professed scepticism. What right, therefore, have they to complain when the world either pities them for their weakness, or abhors them for their wickedness?

Another reason, perhaps, why intellectual scepticism has never prevailed to any considerable extent in the world is, that it is necessarily *suicidal.* By a law of its nature, it no sooner begins to live than it dies—self-destroyed. Does not he who professes to be a sceptic, by that very profession put a dagger in the heart of his scepticism? How can a man *know* himself to be a sceptic? To say that he *knows* that he is such, is to proclaim himself not a sceptic. The real sceptic *knows nothing;* he *doubts every thing;* and therefore, to make any positive assertion about the matter is obviously to misrepresent himself. Hence, no one really believes him, or can believe him. Is it wonderful, therefore, that so few care to stand in such a position?

We trust, then, that it has now been made to appear *how* this universal conviction of a God—all-powerful, just, wise, and good,—*how*, we say, this conviction operates to give wonderful certitude to faith in our faculties. It throws down an illumination so broad, deep, and vivid upon the true nature of man and the foundation of all his knowledge, that even the most simple-minded and the least book-learned can not be insensible to its power. Its influence upon every healthy mind is irresistible, overwhelming. In the light of God's being and character we see, we feel that we have *rock* to rest upon; that man's knowledge, all true science will endure forever, and that life is no dream, but a substantial, solemn reality.

A few words of counsel, and we close this necessarily very imperfect exhibition of a most important subject. First,

1. Always *faithfully employ your faculties*; and when you have in this way arrived at their testimony, treasure it up for future use. You can know nothing in any other way; you can perform well your part in life in no other way. He who does his duty here is gathering the elements of wisdom, usefulness, prosperity, respectability, honor. It is impossible for that man to fail. Again—

2. Our subject, clearly understood, *throws light upon many errors* and *dangerous mistakes*. Take a single one, as an example. How often do we hear unthinking men complain that faith should be the ground of religion, and that only they who come up to it and stand there can be saved. "All this," they say, "is unreasonable, purely arbitrary; that if the evidence for a matter is sufficient, a man can not but believe it; and, on the contrary, if he does not see the evidence to be sufficient, he can not believe it; and must, therefore, perish without any fault on his part."

But need we say that this is altogether a shallow and false view of the subject? The truths of religion, like all other truths, appeal to our faculties as reasonablebeings, and we are called upon to attend to these truths, and then rest upon the testimony of our faculties. In other words, we are to exercise *faith* here just as we do in every other department of knowledge.

But as men can shut their eyes against natural light, and so remain insensible to all the beauty and glory of God's works spread out before them, may they not also close up their minds against truth, and turn away their ears from its lessons? May they not, and alas! do not multitudes here altogether neglect the means of instruction and conviction? We have this fearful power; we may choose darkness rather than light. It is a part of our moral probation.

Faith is thus, to a great extent, a voluntary exercise, and under our control; and therefore, just so far as we refuse to employ it, do we sin against our nature, as well as God's word, and so wilfully walk in the dark, and bring down upon ourselves a just condemnation for our culpable ignorance. Only let these complainers give but a moiety of that attention to the truths of religion and the interests of their souls that they do to the things of this world, and every man of them would speedily become "wise unto salvation through faith which is in Jesus Christ."

It is, then, no peculiarity of religious truth that it demands *faith*, and must be received by faith. All man's knowledge, natural and supernatural—human and divine,—rests, and must forever rest upon faith. He who refuses to exercise confidence in the constitution God has given him—those faculties which God has entrusted to him, and commanded him to use and rely upon,—that man must remain a fool all his days, and perish in his folly.

Here we are prepared, intelligently, to render the tribute of our admiration to one of the greatest minds of antiquity—we mean Augustine, Bishop of Hippo. The schools of philosophy in his day had laid down the principle as of unquestionable truth, "*First knowledge, then faith.*" "No," replied the great African Bishop,—looking far deeper into the philosophy of human nature than any of his time, and anticipating the profounder researches of coming ages—"*No,*" he replied, "FIRST FAITH, THEN KNOWLEDGE."* Finally—

* Aurelius Augustinus was born at Tagaste, a town in Numidia, a Roman province in the north of Africa, Nov. 13, 354 A. D., and died in Hippo

3. Be sure to bring your capacities of intelligence and improvement faithfully to bear upon your *highest interests.*

"Seek first the kingdom of God and the righteousness thereof." This is not only a divine requisition, but one of the clearest dictates of a sound understanding. Never be so absorbed in the things of this life as to forget that you have another and an endless life before you. And the same understanding and the same integrity, prudence and industry that will enable you to succeed in the world, are sufficient, if properly employed, under God's blessing, to secure to you eternal life.

Do not be the victims of the false notion that the blessings of true religion, unlike temporal benefits, come to us in such a mysterious way as supersedes the necessity of human exertion. This is not the teaching of the Bible. It is a miserable delusion of a deceived, depraved heart; and if not shaken off, must prove fatal to the soul. We may never expect any benefit or blessing without seeking for it. This is the great law written not only in God's word, but on the whole constitution of things. It is only "the hand of the diligent that maketh rich" in this world; and so also we must "strive to enter into the strait gate and the narrow way which leadeth to life" eternal. Christianity is one of the most reasonable things in the world. It comes to us just as every other branch of true knowledge does, and as every other valuable attainment does. Its requirements are—"*Prove* all things; hold fast that which is good." "*Press* towards the mark for the prize of your high calling." "Be *diligent* in business, fervent in spirit, serving the Lord."

A simple, but wonderful way is indeed opened up in the

---

Regius, in the same province, Aug. 28th, 430. "He was," says Guericke, the historian, "a man of deep and powerful nature, not the most learned, yet the greatest of the Fathers, and in whose energetic mind acuteness and profundity were blended in their highest degree."

Before the conversion of Augustine, he was deeply imbued with the Manichean Philosophy; and it is against this erroneous system he afterwards most largely and efficiently employed his great principle—"*Fides præcedit intellectum.*"—[NEANDER, VOL. II., p. 359, Prof. Torrey's Ed.]

gospel, by which we may attain, through grace, to the pardon of all our sins, and the regeneration of our whole nature,—body, soul, and spirit,—thus preparing us for a new, holy, useful, blessed life here and the inconceivably higher holiness, usefulness, and blessedness of heaven. But for all these precious and eternal mercies God hath said—" I will be inquired of to do them." And knowing our weakness, and our proneness to unbelief and sin, He has given us for our encouragement promises innumerable—plain, precious promises. Take this one for example—" Ask, and it shall be given you; seek, and ye shall find; knock, and it shall be opened unto you: for every one that asketh, receiveth; and he that seeketh, findeth; and to him that knocketh, it shall be opened. Or what man is there of you, of whom if his son ask bread, will he give him a stone? Or if he ask a fish, will he give him a serpent? If ye then, being evil, know how to give good gifts unto your children, how much more shall your Father who is in heaven give good things to them that ask him?" How clear, full and perfectly reasonable is all this! It is simply—He who would attain to God's favor and eternal life, must sincerely desire it, and earnestly seek it; and none ever yet thus faithfully sought, and failed to obtain.

Yes, we have the assurance of eternal truth, the word of God, who can not lie, to rest upon—" Him that cometh unto me, I will in no wise cast out."

And has Heaven, in love unspeakable, shed down light in such rich abundance upon our path? O let us, then, open all the channels of our souls to receive it! Let us, with faith, quickened and invigorated by the Spirit of God, cordially welcome this light! Only truth, thus admitted and embraced, has power to raise us up to a new and divine life,—power to bring us forth from a dark, sin-enslaved world into the holy liberty of God's dear children—his peculiar people, zealous of good works. And, being thus sanctified and justified through faith in the Lord Jesus and by the Spirit of our God, our path will shine more and more, till swallowed up and lost in the brightness of the perfect day.

